# Miscellaneous Practice

**Published:** 1871-05

**Authors:** F. B. Norcom

**Affiliations:** Chicago


A MONTHLY RECORD OF

*Medicine, Surgery, and the Collateral Sciences.*

EDITED BY J. ADAMS ALLEN, M.D., LL.D.; AND WALTER HAY, M.D.

VOL. XXVIII.—MAY, 1871.—NO. 5.

### Original Communications.

ARTICLE I.—*Miscellaneous Practice.* By F. B. NORCOM, M.D., Chicago.

NERVOUS SYPHILIS.

CASE I. Mr. N——; aged 40; married; nervo-bilious temperament; spare in figure, but of large muscular endowment, and great powers of endurance; placed himself under my charge, July 17th, 1870, afflicted with headache, vertigo, etc. Had no hereditary tendencies, and his habits were passably correct, not being addicted to the use of liquors, or tobacco to any very great extent, and moderate in all other indulgencies. He had always enjoyed excellent health, being free from all diseases, except the eruptive disorders of childhood, and the attack of "break bone" fever a few years back, while residing in the South.

In April, 1855, was attacked, about two weeks after an intercourse, with a bubo of left groin, which, in consequence of his vocation requiring very active exercise, ran rapidly to suppuration. Never was preceded by a sore, though it was carefully searched for, both by his surgeon and himself. The swelling was well laid open, but the peripheral inflammatory exudation was so extensive, that a high degree of constitutional disturbance was engendered, and such a condition of blood deterioration, that a few weeks later several glands of right thigh, below Poupart's ligament, became involved with still further disturbance. It was not until the following September that he could return to his business, though he had been sent into the country, and his treatment —alterative, tonic, and nourishing—had been conducted upon the most approved principles. About June also, two roseoloid patches appeared on his forehead, with moderate nocturnal pains, slight alopecia, and mild faucial erythema, but under the use of bark and mercury, etc., as above mentioned, these symptoms soon disappeared. After the autumn, no further manifestations of a specific character showed themselves, until February, 1856, an ulcer appeared, of a low grade of inflammatory action, on the anterior left leg, which healed, however, in a few weeks, leaving a well marked coppery stain, and pointing decidedly to constitutional contamination.

On application July 17th, fifteen years from his primary attack, he complained of persistent frontal headache of three weeks standing, either increased at night, or the early morning, lessening during the day, but with increasing vertigo, so well marked by noon, as almost to prevent locomotion, often compelling him to sit down, or catch at something for support. Accompanying these, a feeling of oppression at the base of the skull posteriorly; slight impaired power of the tongue; a tired condition of the whole body; a tight, subcutaneous, band-like drawing of the angles of the nares and upper lip, extending to angles of the mouth, and transversely of the calves of both legs; and so much weakness of muscular strength, as to disable him from placing one leg forward of the other in walking, or pressing the foot with security to the ground, thus resembling to a degree the symptoms of incipient locomotor ataxia. Slight numbness of forearms, especially the right, with imperfect extension, including hands and fingers; capricious appetite; coated tongue; face drawn and muddy; no tenderness of head upon palpation, or percussion; and no changes of internal viscera.

Not deeming this a "bilious case," nor attributing these symptoms to a perverted action of that much-abused organ, that "mine of mystery," the liver, which had already been spurred up, and ill used to the extent of most women's wombs now-adays, I came to the conclusion that syphilis was at the bottom of his trouble, in the shape of either a primary congestion leading to beginning pachymeningitis, or what was still more probable, a gummy exudation, vasic and interstitial. Accordingly, fifteen grains iodide potassium was given in cinchona mixture three times a day; warm bathing; nutritious dietary; absolute exclusion from liquor and tobacco; and a large dose of the bromide potassium at night to relieve the brain, if much oppression should be manifested. Under this management, he began slowly to amend, and in the course of a week I ran up his iodide to thirty grains to the dose, with such decided effect as to free him almost entirely from his headache and vertigo, at the same time with amelioration of his other nervous symptoms; but some derangement of digestion, and a copious eruption of acne of face, back and shoulders ensuing, was forced to omit the mid-day dose, and apply a spirituous lotion with the bichloride, and juniper-tar soap to relieve the cutaneous difficulty. In a week or so, the stomach having regained its wonted tone, I pushed the treatment, which was steadily pursued to October, when being entirely relieved of all uneasy sensations of the head, or other parts, and having increased in weight and strength in a decided manner, I gradually withdrew his remedies, and at this moment, nearly nine months from the beginning of his trouble, he is to all appearance in the enjoyment of perfect health.

To very many of my readers, especially those not well versed in syphilis and its many vagaries, this diagnosis may seem far-fetched, and some might suppose that the various nervous phenomena and the systemic accompaniments were significant of many other disorders, functional or otherwise, but a strict review of this case, with the rapid disappearance upon treatment, will in all probability satisfy them that a correct solution was arrived at, and consequently the patient saved from harsher and more dangerous evils. Persistent intercranial pain, increased at night, as first noticed by Moreau, with vertigo, and the band-like feeling of any special set of muscles, (Rollet), together with moderate paresis and impaired power, and the concomitant systemic disturbance, will generally point to specific disease as the causation, the more certainly when the subject has been visited by syphilis several years preceding. Taking the age, general appearance, habits, and total freedom from hereditary taint of this gentleman, one could hardly fall back upon apoplectic plethora, softening, or incipient sclerosis, even though the loss of muscular power in the lower extremities and the uncertainty of gait inclined us to favor this latter disease; but that the pathological lesion indicated either a congestion, or gummata forming at the base, affecting the optic thalami, and those portions of the brain from which originate the facial, fifth pair, glosso-pharyngeal, and the upper spinal cord, the well marked improvement under large doses of the iodide abundantly proves.

The subscribers to this JOURNAL may not have forgotten the case of Mr. B—, No. 2, published in the February number, 1870, where I remark, "on the 11th of August, while walking in the street he was suddenly struck by hemiplegia, being preceded only the night and morning before by some little vertigo, distaste for food, and a feeling of *malaise*. I saw him a few hours after his seizure, found complete palsy, hyperæsthesia, and for a short time increased temperature of the left side, with well-marked reflex movements, no abnormal intercranial sensations, but palsy also of same side of face, tongue, and enlarged pupil."

This occurred, as you will perceive, fifteen months after the initial chancre, *without loss of consciousness*, and suddenly too, without the usual slowly advancing prodromes, and very early in life, his age being only 27 years. Is it possible that the same abnormal conditions of the brain may have existed in this case at such an early date, and that of the one under discussion after as many years? or is it not more probable that Mr. B— was laboring under the primary lesion congestion (perhaps cerebral syphilis *sine materiâ* (?)), without more serious complications, as evinced by the thorough disappearance of all symptoms, after a few weeks saturation by the potassium? Although the first patient was simply oppressed by very disagreeable associates, I have no doubt in my mind, that a few weeks or months later he would have been attacked by paralysis, had not a clearance been effected by prompt and energetic measures. I have the record of several cases of the nervous forms of this disease, paralysis of special sets of muscles, hemiplegia, paraplegia, etc., occurring from six months to twenty years after the first stage, and several of these are dead, and others left in a deplorable state, from the improper and unscientific treatment pursued—the active agent not being recognized—and consequent irreparable injury of nerve-tissue. Possessing a fair experience in the management of such subjects, I can safely recommend that, when the iodide potassium is called for, it be exhibited in very large doses, twenty to thirty grains three times a day to begin with, and no limit be placed, only so far as derangements of stomach or skin may demand an occasional remission. Remember, that in severe cases there exists either a bony or gummy lesion, and if you would ward off destructive changes of nerve-structure, and avert death from your patients, you must use the remedy without regard to quantity, only regarding a favorable amelioration of the symptoms.

Iodism is not to be reckoned; and mercury, no matter how guarded, or combined, to be shunned; but to render efficacious any form of specific treatment, such essentials as pure air, nourishing food, regular sleep, tonics, etc., must be considered, and such measures must be perseveringly followed many months after a perfect cure has apparently taken place.

### GUTTA ROSACEA.

(Synonyms :—Bacchia ; Rosy Drop ; Brandy Face ; *improperly* Acne Rosacca.)

CASE 2. Mrs. S——; aged 30; married, and mother of one child; nervo-sanguineous temperament; came under my treatment Oct. 8th, 1870, afflicted with gutta rosacea of five years standing. She possessed no hereditary proclivities, coming from very healthy stock, and had never suffered from any severe illness, even excepting those of younger life. At the age of fourteen years, was visited by an attack of acne punctata et comedones, aggravated during the menstrual epochs, and which continued off and on until her marriage, then growing less and less, and finally disappearing to give place to her cutaneous ailment, one year anterior to the birth of her child, now four years of age.

This affection began simultaneously on the prominences of both cheeks, first by flushes, induced probably by the stimulus of food and mental emotions, and from this erythematous stage, gradually, through a succession of months, assuming higher inflammatory types, until its present condition. Upon a strict examination, I found two extensive patches, one on each cheek, presenting several phases of this well known disease; the central portions, evidently the longest exposed to inflammatory congestion, exhibiting those varieties, known as gutta rosacea tuberculosa, and pustulosa; while toward the circumferences, the milder forms came under our observation, such as punctata and papilosa. The skin was thickened to some extent by inflammatory exudation, and she experienced at times, as almost from the first, the disagreeable sensation of burning, itching, and latterly severe throbbing pains. Apart from these subjective symptoms, the objective disfigurement had rendered her extremely unhappy; debarring her in a great measure from society, unless among her intimate friends; causing great mental despondency; and thus reacting upon her general health, creating marked nutritive and nervous debility; while, to cap the climax, as it were, in the fifth year of her trouble, the nose began to exhibit signs of capillary stagnation, and at this moment is in a state of blooming hyperæmia.

The roseate hue, and the uneasiness of this eruption, are much enhanced by full meals, especially of stimulating food, or condiments; by exposure to a heated atmosphere; or by the inevitable reaction following exercise in the cold air; and though she has submitted herself to various forms of treatment under several physicians, she seemed hardly to have derived even partial benefit, and in several instances suffered such excruciating torture from the local remedies used, that she was forced to abandon them, and at the same time all hopes of a deliverance from this severe visitation.

Her mental distress may be well imagined, when I state that vanity is a characteristic trait of the human family,—not of the female portion exclusively, as most people would ungallantly urge, for, there is no person on the face of the globe with a larger share of this ingredient than the biped man, and the uglier, and more forbidding, the more he has of it,—but, at the same time in the fairer sex it is a pardonable quality, as it leads to the adornment of their persons, the preservation of their beauty, and the envy of their rivals (especially intimate and confidential friends,) all for the sake of pleasing and attracting the *soi-disant* lords of creation, and one can readily excuse and pity any fair lady, who may be subjected to such defacement of her personal appearance.

The diagnosis should not be at all difficult, since few diseases of the skin can be mistaken for it; mayhap lichen, impetigo, or the erythematous or papilous eczema, but the general history, the

*locale* and the age will clear up all doubt. It very frequently is miscalled acne rosacea, a very old name, and placed, in the classification of several authors, among the pustulæ; but acne is essentially a disorder of torpid glandular secretion, leading to the accumulation of sebaceous matters, and by mere mechanical pressure, causing irritation and inflammation. It belongs to the eczematous family, and has a constitutional origin; non-contagious, chronic, but amenable to treatment, though it may have to be pursued perseveringly many months or years. Recognizing the existence of nervous and nutritive debility as a definite constitutional element, I began by prescribing the arsenio-ferruginous mixture to give tone to the system, and reinstate her general health; at the same time allowed her a nutritious dietary, excluding crude or indigestible materials; regulated the secretions; advised warm bathing, and exercise in the open air; and all the moral suasion in my power, to calm her mental disturbance, by the assurance of a certain cure, without painful applications, or nauseating medicines. Locally, a wash of the bichloride and spirits of wine; the benzoated oxide of zinc ointment; and several times a day an ablution of cold water, with juniper-tar soap. This treatment once begun was persisted in steadily, increasing from time to time the arsenic, until about ten drops of Fowler's Solution three times daily were reached; and an occasional change of the ointment, to that of the compound hypochloride of sulphur. Of course, she was cautioned against every thing that might act as a remote predisposing cause to perpetuate this disease, and as I could detect no direct one, excepting constitutional *debility*, my measures were mainly directed to overcome this, and with such effect, too, that in three months the skin had thinned very materially, and almost completely lost its characteristic features.

She is still taking the iron and arsenic, and obeying my directions in every particular, and though the last time I saw her, late in March, with a total subsidence, if we except the congestion of the nose, she will continue the remedial measures increasingly for at least a year, or perhaps more.

It may seem superfluous to comment further on this case, as belonging to that class of cutaneous affections, known as chronic, and for which very few among our profession have any liking, unless indeed they make special study of the same; yet I do not wish to leave before reiterating my views on the exhibition of arsenic, and giving some caution in regard to the topical application of remedies. It is well understood that one of the greatest authorities on such subjects—Wilson—rarely prescribes arsenic except in small doses, continues it for years if necessary, and never desires to obtain its decided physiological effects. But my own experience has forced me many times to increase this agent to very large quantities, until in fact systemic irritation was induced, before I could possibly see the least benefit resulting. I feel certain that, in many chronic cases, the remedy has been used month after month, until the patience of both the afflicted and the physician were exhausted, and it was only after its poisonous qualities—if they may be termed such—were manifest, that improvement rapidly began, and if I am not greatly mistaken, this is the view held by many experienced men, and now certainly gaining ground. I can only add, that if those interested will try it, they will not be disappointed.

Again, it is entirely unnecessary, in making local applications to the great majority of such affections, that pain be produced; it is a great drawback to the cure as a rule, occasioning local irritation. if not inflammation, creating often foreign complications, and disheartening your patients. The more soothing you can make your applications consistent with their curative power, the more certainly they will fill your expectations, and the more the unfortunates will appreciate your skill and forethought in their behalf.

### ASPERMATISM.

Case 3. Ben, a griff negro; aged 34 years; married; with large muscular development; came to consult me May, 1863, in regard to an inability to perform the marital act, saying he had "lost all ambition"; a lamentable circumstance, viewed by him, as by every other negro, in the light of a dreadful calamity, and as foreshadowing the end of the world, or perhaps the coming of the millenium. His antecedent history was that of robust health, and exemption from disease, with the exception of occasional attacks of intermittent fevers, diarrhœa, or catarrh, lasting generally a short time, and imparting no weakness to his constitutional vigor.

The history of his present trouble dated some three months back, about the middle of February, when the fact gradually dawned upon his startled intelligence that, though perfect in erection, there was too much hard work—as he termed it—and too great an expenditure of breath to bring about the orgasm; and as time waned on, the imagination being now thoroughly aroused, matters grew worse and worse, until the consummation became absolutely impossible, though possessing the working power of a six-horse engine; and to complete his misery, erotic dreams were introduced several times a night, with spermatic floodings, which he looked upon with disfavor, as an unsatisfactory appropriation, and a dead loss of so much of the raw material. At the date of his application, he was apparently in the enjoyment of good health, and a strict examination of the sexual organs, and their associates, elicited nothing determinate or satisfactory; scrotum very flaccid, about the size of a half-peck sack, and enclosing two ponderous testicles, a little tender upon palpation; penis, enormous in length and size, bearing a close resemblance to a member of the ophidion genus—known as the coluber constrictor—without stricture; and bladder and prostate natural. Subjectively, he complained of weakness in the loins; paroxysmal pains in the testicles, especially after the nocturnal orgasms; deficient appetite; and a melancholic feeling, as if life were a delusion, and stripped forever of all enjoyment; yet clung to that blessed boon, hope—still left us all in the bottom of that mythological box—to cheer him on to eventual recovery. As I had never before encountered such a case, my diagnosis was necessarily obscure and faulty; it was no priapism, nor erotomania, nor impotency, in the proper acceptation of this term, and thus reasoning by exclusion, I was forced to fall back upon aspermatism, of which I knew very little, and hence could hardly expect that any treatment based upon such imperfect knowledge would meet with deserved success.

Supposing the pathological condition to be that of abnormal irritation of the muscular endowment of the ejaculatory ducts, arising, doubtless, in this subject from inordinate venereal indulgence, I began by establishing repeated counter-irritation in the perinæum by vesication, the size of a sixpence, and dressing with belladonna ointment; full narcotic of opium at night; white mixture to regulate the bowels; digestible, but unirritating, food; cold bathing to the loins; total abstinence from liquors, of which he was very fond, and of females, of whom he was much fonder; and large doses of chlorate of potass, for what particular indication, I could not say. This treatment, so far I as could control the patient, was insisted upon vigorously, not only for the sake of science, but to secure a cure as soon as practicable, in order to allay any feeling of displeasure in the community, for Ben was a gay Lothario, (a kind of "plantation bull,") and in great request among the dusky Phrynes of the neighborhood. That he did not obey me strictly in regard to "wine and women" is pretty cer- ain, and that he neglected the other measures is equally true, but before the end of August the disease was under full subjection, and he reported himself a man again, and able to hold his own, "widout loss of bref," and "widout breakin' hissef all to pieces."

During the management of this boy, I consulted all the literature on the subject, not only in my own possession, but the libraries of my friends, and discovered how very few and scattering were such cases, one here and there among the medical journals, finding, at length, that excellent treatise on "Impotency and Sterility" by Routand, a thoroughly scientific *brochure* published in Paris about the year 1855 or 1856, containing the histories of several such, and to which he was the first—if I am not mistaken—to give the name of "Aspermatism," a misnomer, however, since there is no deficiency of seminal secretion, but simply a want of power to eject, occasioned by spasmodic contraction of the muscles of the ejaculatory canals, and their consequent occlusion, thus preventing the completion of the act.

Within the last year, another case, but by no means so well marked, presented itself in the form of a gentleman suffering from gleety discharge, unaccompanied by stricture.

There was considerable irritation of the distal portion of the urethra, as was evinced by passing the sound, some tenderness of the prostate gland, and slight aching, at times, of the testicles. Lascivious dreams, with emissions, were frequent, often two or three times a week, with occasional remissions of two or more nights, and it was only at long intervals—once a month or so—that he could succeed at all in sexual intercourse, though the erections were perfect, but now and then painful to priapism. He gained very materially under the passage of steel instruments twice a week, with large doses of bromide of potassium at night; belladonna and iron twice a day; cold salt bathing of loins and pelvis, etc., but being obliged to leave the city after a few weeks for the far West, I have been unable to follow up the record. It seems difficult to account for this peculiar condition pathologically, except upon the assumption of direct or reflex irritation, determined, through constitutional idiosyncracies, by an inflammatory state of any or some portion of the urethral track, or neighboring organs, but as my own information and experience were extremely limited, I was inclined to doubt whether the treatment was consistently philosophical, or had any influence whatever in mastering this condition. Now, Ben was under supervision, more or less, for three months, though the counter-irritation and belladonna were not persisted in longer than six or seven weeks, and it is highly probable that the other measures were also inefficiently carried out; but he began to improve markedly, and when once this change for the better took place, there was very rapid advance, and never retrogression. The balm to his disturbed mind, too, in my assurance of his final recovery—a prognosis which I had no right to give, and which, in my ignorance, I had no right to predict—may have had as much weight in the scale as the more material remedies exhibited.

